# Immunomodulating Therapies in Breast Cancer—From Prognosis to Clinical Practice

**DOI:** 10.3390/cancers13194883

**Published:** 2021-09-29

**Authors:** Marcus Schmidt, Anne-Sophie Heimes

**Affiliations:** Department of Obstetrics and Gynecology, University Medical Center Mainz, 55131 Mainz, Germany; anne-sophie.heimes@unimedizin-mainz.de

**Keywords:** tumor infiltrating lymphocytes (TILs), immune checkpoint inhibitors (ICPis), mRNA vaccine, tumor-associated antigens (TAA), neoantigens

## Abstract

**Simple Summary:**

The role of the immune system in breast cancer has been debated for decades. It is generally accepted that tumor-infiltrating lymphocytes are associated with positive prognostic and predictive effects, especially in triple negative breast cancer. This subset of breast cancer is characterized by the absence of hormone receptors and human epidermal growth factor receptor 2. Compared to other breast cancer subtypes, triple-negative breast cancer has more mutations and neoantigens, making it more immunogenic. Releasing the brakes on the immune system with the help of so-called immune checkpoint inhibitors leads to activation of the immune system and destruction of cancer cells. This, in turn, improves survival, especially in early and advanced triple-negative breast cancer. A new and promising strategy is the enhancement of the immune response using individualized mRNA vaccines against tumor-specific neoantigens.

**Abstract:**

The role of the immune system in breast cancer has been debated for decades. The advent of technologies such as next generation sequencing (NGS) has elucidated the crucial interplay between somatic mutations in tumors leading to neoantigens and immune responses with increased tumor-infiltrating lymphocytes and improved prognosis of breast cancer patients. In particular, triple-negative breast cancer (TNBC) has a higher mutational burden compared to other breast cancer subtypes. In addition, higher levels of tumor-associated antigens suggest that immunotherapies are a promising treatment option, specifically for TNBC. Indeed, higher concentrations of tumor-infiltrating lymphocytes are associated with better prognosis and response to chemotherapy in TNBC. An important target within the cancer immune cell cycle is the “immune checkpoint”. Immune checkpoint inhibitors (ICPis) block the interaction of certain cell surface proteins that act as “brakes” on immune responses. Recent studies have shown that ICPis improve survival in both early and advanced TNBC. However, this comes at the price of increased toxicity, particularly immune-mediated toxicity. As an alternative approach, individualized mRNA vaccination strategies against tumor-associated neoantigens represent another promising approach leading to neoantigen-specific immune responses. These novel strategies should help to improve treatment outcomes, especially for patients with triple negative breast cancer.

## 1. Introduction

Breast cancer is the most common cancer and the leading cause of cancer death for women worldwide [1]. In 2015, breast vancer incidence was 2.4 million, with 523,000 breast cancer deaths. Invasive breast cancer can be divided in several molecular subgroups (e.g., luminal A, luminal B, HER2-positive, and triple-negative) which have different prognoses and different systemic therapeutic options (e.g., chemotherapy, endocrine therapy, anti-HER2 therapy) [2]. Early breast cancer has no distant metastases and is curable [2]. However, if distant metastases occur, the disease is treatable but incurable [3].

The role of the immune system in breast cancer has long been debated [4]. With the advent of modern techniques, such as mRNA sequencing data from The Cancer Genome Atlas (TCGA), it has been shown that high expression of T-cell and B-cell signatures predicts improved overall survival in many tumor types, including breast cancer [5]. In particular, triple-negative breast cancer (TNBC), which has a more pronounced immunogenic potential compared to other molecular subtypes, is of great interest. TNBC accounts for up to 20% of breast cancers and is associated with a significantly worse prognosis in the first 2 to 3 years after diagnosis compared with other breast cancer subtypes [6]. It is now generally accepted that TNBC is not a homogeneous disease. Instead, TNBC consists of multiple subtypes (e.g., basal-like 1 and 2, immunomodulatory, mesenchymal, mesenchymal stem-like, and luminal androgen receptor) [7]. In a comprehensive immunogenomic analysis of over 10,000 tumors using TCGA data, Thorsson and co-workers identified six stable and reproducible immune subtypes C1–C6 (i.e., wound-healing, IFN-γ-dominant, inflammatory, lymphocyte-depleted, immunologically quiet, and TGF-β-dominant) [8]. Interestingly, these immune subtypes include multiple tumor types, and are characterized by a dominance of either macrophage or lymphocyte signatures, T-helper phenotype, extent of intratumoral heterogeneity, and proliferative activity. Although these authors did not comment specifically on TNBC, it is likely that TNBC with a strong lymphocytic infiltrate belong to immune subtype C3. Using even more sophisticated techniques, such as single-cell sequencing, Wu and his collaborators have deconvoluted breast cancer cohorts and stratified them into nine clusters, called “ecotypes”, with unique cellular compositions and clinical outcomes that provide a comprehensive transcriptional atlas of breast cancer cellular architecture [9]. Significantly more somatic mutations and neoantigens are detected in TNBC than in other molecular subtypes, resulting in increased immunogenicity [10]. In a systematic review, Stanton and colleagues showed that the extent of tumor-infiltrating lymphocytes (TILs) varies within and between breast cancer subtypes, with TNBC having numerous TILs [11]. This may identify breast cancers that are more suitable for immunotherapy.

## 2. Brief Overview of the Immune System in Breast Cancer

**Figure 1:**
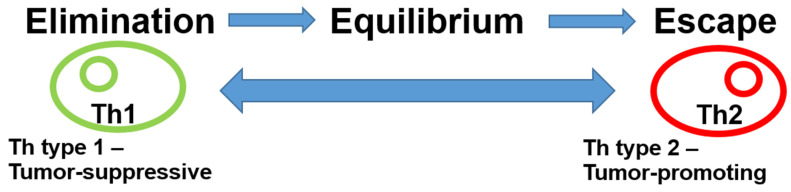
Immunoediting in breast cancer. Cancer immunoediting has dual host-protecting and tumor-sculpting actions of the immune system that not only prevent but also shape neoplastic disease. Cytokines determine T-cell polarization. Th1 and Th2 cells are characterized by their mutually exclusive expression patterns of cytokines. Th1 cells produce IFN-γ, whereas Th2 cells produce IL-4, IL-5, and IL-13. The presence of IL-12 and/or interferon-γ drives previously uncommitted T cells to become polarized to produce T1 cytokines, while IL-4 drives them to become polarized to secrete T2 cytokines. This, in turn, leads to either a tumor-suppressive or a tumor-promoting microenvironment. Abbreviations: Th, T helper cells.

The role of the immune system in the breast cancer microenvironment is ambiguous. Following the presentation of antigens by antigen-presenting cells (APCs), various immune system responses may occur. On the one hand, tumor-inhibitory acute inflammation may develop, driven by type 1 T helper cells (Th1) via CD8 lymphocytes, B cells, or M1 macrophages. On the other hand, tumor-promoting Th2-driven chronic inflammation can also occur through M2 macrophages, regulatory T cells (Tregs), or immune checkpoints, such as programmed cell death protein 1 (PD-1) or its ligand programmed cell death 1 ligand 1 (PD-L1). In addition, bone-marrow-derived cells, such as myeloid-derived suppressor cells and mesenchymal stromal cells, can exert pro-tumorigenic effects through negative regulation of immune responses. Originally, it was thought that Th1 and Th2 cells are characterized by their mutually exclusive expression patterns of cytokines. Th1 cells produce IFN-γ, whereas Th2 cells produce IL-4, IL-5, and IL-13 [12]. Recent results, however, have shown that a single-cytokine-based nomenclature fails to capture the complexity and diversity of T helper cells [13]. Immunoediting, the dynamic interaction between the immune system and the tumor, leads to different stages of tumor evolution (elimination–equilibrium–escape) [14,15]. This process is responsible for both eliminating tumors and sculpting the immunogenic phenotypes of tumors that eventually form in immunocompetent hosts in the early phase of breast cancer development. The acute inflammatory response creates a T helper (Th) type 1 microenvironment at the tumor site, leading to an immune response that is tumor-suppressive and destroys tumor cells. Tumor cell variants, however, can escape the immune response. This immunoediting creates a state of equilibrium. When inflammation transitions from acute to chronic, a Th type 2 profile develops, leading to tumor-promoting effects with escape from the immune system and uncontrolled tumor growth (Figure 1).

## 3. Prognostic and Predictive Significance of Tumor-Infiltrating Lymphocytes

Most studies that addressed the prognostic and/or predictive role of TILs in breast cancer focused on the cellular immune system, particularly cytotoxic T cells [16,17,18,19,20,21]. Overall, these studies showed that increased rates of tumor-infiltrating lymphocytes or T-cell transcripts were associated with improved prognosis in rapidly proliferating breast cancer such as TNBC.

In contrast, we primarily examined B cells and the humoral immune system and reported a strong positive prognostic impact of a B cell metagene on breast cancer prognosis [22]. This strong protective effect of a B cell/plasma cell signature was later confirmed by others [23,24]. Tumor-infiltrating plasma cells were identified by confocal microscopy as the source of immunoglobulin kappa C (IGKC) expression [25]. In this study, co-staining with anti-human IgG showed that IGKC was expressed in IgG-positive cells, a known feature of B-cell maturation and plasma cell differentiation after antigen contact. IGKC has been associated with favorable prognosis in untreated patients and with response to anthracycline-containing neoadjuvant chemotherapy in early breast cancer [25]. Indeed, in a comprehensive analysis of the prognostic landscape of genes and infiltrating immune cells in human cancers, Gentles et al. confirmed that plasma cell signatures, as well as plasma cells expressing IGKC, are associated with improved survival [24]. However, the strong dependence of the humoral immune system on T cells is examplified by C-X-C motif chemokine ligand 13 (CXCL13)-positive CD4+ follicular helper T (Tfh) cells, which are crucial for germinal center development and antigen-specific B cell maturation to high-affinity memory cells and antibody-secreting plasma cells [26]. In addition, CXCL13 has been associated with improved survival in TNBC [27].

Overall, these and other findings suggest that humoral immunity may be as important as cellular immunity in eliminating cancer [28]. These, initially retrospective, results were later confirmed in exploratory studies using archival tissue from randomized trials [27,29,30], as well as by histological evidence of TILs in archival tissue from randomized trials [19,31,32]. Recently, in the neoadjuvant EXPRESSION trial, we demonstrated that genes with significantly higher expression in pathologically complete responders are primarily related to the immune response, including immunoglobulins [33]. These results also support the predictive role of the humoral immune system in early breast cancer.

Particularly in triple-negative breast cancer, there is a strong association between TILs and a more favorable prognosis or response to neoadjuvant chemotherapy [31,32,34,35,36,37]. Overall, increased tumor-infiltrating lymphocytes in TNBC resulted in increased complete pathologic response (pCR) and also improved survival (Table 1).

This significant association of tumor-infiltrating immune cells and TNBC is not surprising, considering that the overall mutational burden is highest in TNBC [10]. In addition, these authors found that mutational burden was highly correlated with neoepitope load (R2 = 0.86). A comprehensive analysis of immunogenic signatures in TNBC based on two sets of large-scale breast cancer genomic data showed that TNBC has the strongest immunogenicity among breast cancer subtypes [38]. Furthermore, these authors confirmed that TNBC also has higher levels of immune cell infiltration and higher expression of genes encoding immune checkpoints than non-TNBC. However, mutational and neoantigen load appear to incompletely explain the immune response in TNBC, as other studies have described an inverse relationship between immune infiltration and somatic copy number alterations [39,40]. Obviously, the exact relationship between immune infiltration, mutation burden, and neoantigen burden has not been fully elucidated. Nevertheless, TILs are widely used, especially in TNBC. To improve reproducibility, a standardized method for the evaluation of TILs has been defined to integrate this parameter into standard histopathological practice [41,42].

## 4. Immune Checkpoint Inhibitors

Important target structures in the immune system are “immune checkpoints”. Immune checkpoint inhibitors (ICPis) block the interaction of certain cell surface proteins that serve as “brakes” on immune responses. Currently, the most important immune checkpoint in breast cancer is the PD-1/PD-L1 axis [43,44]. The interaction between PD-1 and its ligand PD-L1 functions as an immune checkpoint against unrestrained cytotoxic T effector cell activity. Furthermore, it promotes peripheral T effector cell exhaustion and conversion of T effector cells to immunosuppressive Tregs [45]. Immune checkpoint inhibitors that block the PD-1/PD-L1 axis and reactivate cytotoxic T effector cell function increase immune cell activity against tumor cells.

Indeed, monoclonal antibodies, so-called immune checkpoint inhibitors, which block either PD-1 or PD-L1 (e.g., atezolizumab, durvalumab, nivolumab, or pembrolizumab) are increasingly used to release the “brake” of the immune system and, thus, increase the activity of the immune system against the tumor. A potential problem is that ICPis require the presence of effector immune cells in the tumor, suggesting a baseline immune response to trigger pre-existing immunity [46]. The monoactivity of ICPis such as atezolizumab or pembrolizumab was modest in phase I trials in advanced and extensively pretreated TNBC [15]. Few, but long-lasting, responses were observed, particularly in less extensively pretreated patients [47]. In a phase III trial (KEYNOTE-119) in pretreated advanced TNBC, monotherapy with pembrolizumab did not significantly improve overall survival compared with chemotherapy, although the effect of pembrolizumab treatment increased with increasing PD-L1 positivity [48]. However, the efficacy can be significantly increased by adding chemotherapy. Indeed, chemotherapy may lead to immunogenic cell death, which in turn activates the antitumor immune response [49,50]. The combination of immunotherapy and chemotherapy should, therefore, achieve an additive or synergistic clinical effect [51].

Due to the specific mode of action of immunotherapies, which, in contrast to cytotoxic chemotherapy, have no direct effect on tumor cell proliferation, a therapeutic response can only be expected at a later stage. In addition, infiltration of immune cells may lead to an initial enlargement of metastases, a so-called pseudoprogression [52]. However, this pseudoprogression occurs in less than 10% of cases, whereas, conversely, a very rapid increase in size, known as hyperprogression, may be more common occuring in up to 29% [53]. Therefore, continuation of therapy with ICPi in the presence of imaging evidence of progression should only be considered if the clinical condition has improved and no treatment-related toxicities are present [54].

### 4.1. ICPi in Advanced Breast Cancer

The monoactivity of ICPis such as atezolizumab or pembrolizumab in advanced TNBC has been evaluated in several phase I and phase II studies (Table 2). Depending on PD-L1 status and line of therapy, objective response rates (ORR) ranged from 5.3% to 24%. Progression-free survival (PFS) ranged from 1.4 to 2.1 months and overall survival (OS) ranged from 9.0 to 18 months [47,55,56,57]. Winer and colleagues compared pembrolizumab with chemotherapy for second- or third-line treatment of patients with metastatic triple-negative breast cancer [58]. Randomization was stratified by PD-L1 tumor status (combined positive score [CPS ≥ 1] vs. negative [CPS < 1]) and history of prior neoadjuvant or adjuvant treatment vs. de novo metastatic disease at initial diagnosis. The median overall survival in patients with a PD-L1 CPS of 1 or more was 10.7 months in the pembrolizumab group and 10.2 months in the chemotherapy group (hazard ratio [HR] 0.86; 95% confidence interval [95% CI] 0.69–1.06). The efficacy of pembrolizumab increased with higher CPS. However, no significant improvement was observed.

The above-mentioned studies enrolled patients with advanced TNBC. Very few early-phase clinical trials have included advanced hormone receptor (HR)-positive and HER2-negative patients. Either with an ICPi alone or in combination, the survival was modest [49,50,51,52].

It is obvious that further combinations are needed to increase the efficacy of ICPi in breast cancer. For instance, low-dose metronomic chemotherapy with cyclophosphamide reduced regulatory T cells (Tregs) and enhanced anti-tumor activity of cytotoxic T cells [59]. In addition, vinorelbine, cyclophosphamide, and 5-FU had significant preclinical effects on circulating and tumour-infiltrating immune cells and could be used to obtain synergy with anti-PD-L1 [60]. Furthermore, a combinatorial therapy in preclinical models of breast cancer increased checkpoint inhibition by activating antigen-presenting cells, enhancing intratumoral CD8+ T cells, and increasing progenitor exhausted CD8+ T cells [61]. Radiation therapy also has immunomodulatory effects that could contribute to increased efficacy of immunotherapies [62].

Although preclinical models provide a solid basis that certain low-dose chemotherapies, such as cyclophosphamide or vinorelbine, improve anti-PD-L1 activity in breast cancer, these therapeutic approaches need to be tested in clinical trials. In the adaptive, non-comparative phase II TONIC trial, Voorwerk and coworkers investigated multiple strategies (e.g., radiation, low-dose cyclophosphamide, cisplatin, doxorubicin) to make the tumor microenvironment more sensitive to PD-1 blockade with nivolumab in 67 patients with advanced TNBC [63]. In the entire cohort, the objective response rate (ORR) was 20%. Most responses were seen in the doxorubicin (35%) and cisplatin (23%) cohorts. Interestingly, the authors noted upregulation of immune-related genes in these two cohorts and speculated that this induction approach may induce a more favorable tumor microenvironment and increase the likelihood of response to PD-1 blockade in TNBC.

Another approach to increase the efficacy of an immune checkpoint blockade in advanced cancer is to combine it with poly(ADP-ribose) polymerase inhibitors (PARPi). PARPi-mediated unrepaired DNA damage modulates the tumor immunological microenvironment through a number of molecular and cellular mechanisms, such as increasing genomic instability, immune pathway activation, and PD-L1 expression on cancer cells, which could promote responsiveness to ICPis [64]. In the MEDIOLA basket study, durvalumab and olaparib were combined in solid tumors [65]. This combination showed promising antitumor activity and safety. Disease was under control at 12 weeks in 24 of 30 evaluable patients (80%). While the above combinations are interesting and promising, combining ICPi with chemotherapy is currently the simplest approach. A phase Ib study evaluated the safety and clinical activity of atezolizumab in combination with nanoparticulate albumin-bound (nab)-paclitaxel in a cohort of 33 extensively pretreated patients with advanced TNBC [66]. The rationale for combining an immune checkpoint inhibitor with chemotherapy was the postulated greater activation of T-cell-mediated immunity due to increased release of tumor-associated antigens and resulting immunogenic cell death [51]. The results of the study demonstrated that the combination of ICPi and nab-paclitaxel is an effective treatment option with a tolerable side effect profile for patients with metastatic TNBC (Table 2).

**Table 2:** Phase I/II evidence for ICPis in advanced breast cancer.

Author	*n*	Therapy	AE	ORR	PFS	OS
Emens et al., 2019 [56]	116 TNBC	Atezolizumab	63%	24%	1.4 m	17.6 m
Nanda et al., 2016 [57]	31 TNBC	Pembrolizumab	66.3%	18.5%	1.9 m	11.2 m
Adams et al., 2019 [55]	170 TNBC	Pembrolizumab	60.6%	5.3%	2.0 m	9.0 m
Adams et al., 2019 [47]	84 TNBC	Pembrolizumab	63.1%	21.4%	2.1 m	18.0 m
Adams et al., 2019 [66]	33 TNBC	Atezolizumab + nab-paclitaxel	100%	39.4%	5.5 m	14.7 m
Barroso-Sousa et al., 2020 [67]	8 HR+	Pembrolizumab + radiotherapy	87.5%	0	1.4 m	2.9 m
Pérez-García et al., 2020 [68]	44 HR+	Pembrolizumab + eribulin		18%	6.0 m	-
Yuan et al., 2021 [69]	23 HR+	Pembrolizumab + palbociclib + letrozole		56%	25.2 m	36.9 m

Abbreviations: AE, adverse events; HR, hormine receptor; ICPis, immune checkpoint inhibitors; m, months; ORR, objective response rate; OS, overall survival; PFS, progression-free survival; TMBC, triple-negative breast cancer; vs., versus.

Building on these encouraging results, the phase III IMpassion130 trial confirmed the clinical efficacy of atezolizumab in combination with nab-paclitaxel as a first-line therapy in a cohort of 902 patients with metastatic or locally advanced TNBC [70]. Patients were randomized 1:1 to either the experimental arm (atezolizumab in combination with nab-paclitaxel) or the placebo arm (nab-paclitaxel + placebo). The results showed a significant prolongation of PFS in both the intention-to-treat (ITT) population and the PD-L1-positive subgroup: PFS was 7.2 months in the experimental arm compared with 5.5 months in the placebo arm (HR 0.80; 95% CI 0.69–0.92; *p* = 0.002). In the subset of PD-L1-positive (≥1% of immune cells) TNBC patients, PFS was 7.5 months compared with 5 months in the placebo arm. Atezolizumab in combination with nab-paclitaxel prolonged OS in PD-L1-positive patients (25.0 versus 15.5 months). Based on these results, atezolizumab in combination with nab-paclitaxel is now approved as a first-line therapy for advanced PD-L1-positive TNBC. In a recent IMpassion130 update, Schmid et al. showed that atezolizumab did not significantly increase OS in the overall cohort from 18.7 to 21 months at longer follow-up (HR 0.86; 95% CI 0.72–1.02; *p* = 0.078) [71]. However, in PD-L1-positive patients, OS increased from 18 to 25 months (HR 0.71; 95% CI 0.54–0.94). Surprisingly, the recently presented IMpassion131 trial combining atezolizumab with conventional paclitaxel in advanced TNBC did not improve PFS or OS compared with placebo + paclitaxel [72]. Of 651 randomized patients, 45% had PD-L1-positive aTNBC. In the PD-L1-positive population, atezolizumab and paclitaxel were associated with a more favorable unconfirmed overall response rate (63% vs. 55% for placebo–paclitaxel) and a longer median duration of response (7.2 and 5.5 months, respectively). Possible reasons for this apparent contrast with the benefit observed in IMpassion130 require further investigation, although several hypotheses (e.g., different taxanes and the role of steroids or imbalances in prognostic features or random results in a relatively small study) are under discussion [72,73].

Recently, Cortes and coworkers presented the results of KEYNOTE-355, a randomized, double-blind, phase III trial of pembrolizumab + chemotherapy versus placebo + chemotherapy in previously untreated, locally recurrent, unresectable, or metastatic triple-negative breast cancer [74]. They showed that pembrolizumab in combination with multiple chemotherapy partners (nab-paclitaxel, paclitaxel, or gemcitabine/carboplatin) produced a statistically significant and clinically meaningful improvement in PFS compared with chemotherapy alone in patients with previously untreated locally recurrent unresectable or metastatic TNBC whose tumors expressed PD-L1. Compared with TNBC, there are few randomized data in hormone receptor (HR)-positive/HER2-negative advanced breast cancer. Tolaney et al. randomized 88 heavily pretreated patients with advanced HR-positive/HER2-negative breast cancer to eribulin +/− pembrolizumab in one study. [75]. However, the addition of pembrolizumab to eribulin did not improve PFS or OS compared to eribulin alone in either the intention-to-treat or PD-L1-positive populations. The results of randomized trials in advanced TNBC are summarized in Table 3:

### 4.2. ICPis in Early Breast Cancer

As a result of the efficacy of ICPis in advanced breast cancer, trials have also been initiated in early TNBC. In a randomized phase II trial in early TNBC (GeparNuevo), the PD-L1 antibody durvalumab was combined with anthracycline- and taxane-containing neoadjuvant chemotherapy (NACT) in 174 TNBC patients [76]. In total, 87% of patients were PD-L1 positive. Pathological complete remission (pCR) was increased with durvalumab from 44.2% to 53.4%. A significant increase in pCR was seen in the subgroup (*n* = 117) that received durvalumab neoadjuvantly for 2 weeks before starting NACT (61% vs. 41.4%; *p* = 0.035). Immune-mediated thyroid dysfunction occurred in 47%, with good overall tolerability. Interestingly, a preplanned exploratory analysis of this study showed that both tumor mutational burden (TMB) and immune gene expression profile (GEP) independently predicted pCR in TNBC patients [77]. In patients with high TMB and GEP, the pCR rate was 82% compared to 28% in the low TMB and GEP group. These results encourage further analysis of TMB in combination with immune parameters to tailor therapies in breast cancer. In GeparNuevo, Sinn and coworkers also examined mRNA signatures to predict response to neoadjuvant PD-L1 inhibition in combination with chemotherapy in early triple-negative breast cancer [78]. They found that immune-associated signatures related to antigen presentation and interferon signaling were associated with pCR after chemotherapy, but may be of limited utility in predicting response to additional immune checkpoint blockade. Recently, at the Annual Meeting of the American Society of Clinical Oncology, Loibl et al. presented the long-term survival results of GeparNuevo [79]. Of note, durvalumab was discontinued after surgery. As reported in the primary analysis, durvalumab failed to significantly increase pCR rates. However, 3-year iDFS was 84.9% with durvalumab versus 76.9% with placebo (HR 0.54, 95% CI 0.27–1.09, *p* = 0.0559), 3-year DDFS was 91.4% versus 79.5% (HR 0.37, 95% CI 0.15–0.87, *p* = 0.0148), and 3-year OS was 95.1% versus 83.1% (HR 0.26, 95%CI 0.09–0.79, *p* = 0.0076). The authors concluded that durvalumab as an adjunct to neoadjuvant chemotherapy in TNBC significantly improved long-term outcome despite a small increase in pCR and no continuation after surgery. They raised the obvious question of whether adjuvant therapy with ICPis is necessary at all. Furthermore, in addition to standard taxane- and anthracycline-based NACT, pembrolizumab was studied in the adaptive randomized phase II I-SPY trial [80]. In TNBC, pembrolizumab increased pCR from 22% to 60% with an acceptable safety profile.

In the neoadjuvant phase III KEYNOTE-522 trial, 1174 patients with early-stage TNBC were treated neoadjuvantly with anthracycline-, taxane-, and platinum-containing chemotherapy +/− pembrolizumab [81]. The addition of pembrolizumab significantly increased pCR from 51.2% to 64.8% (*p* = 0.00055). This increase in pCR was observed regardless of PD-L1 status. In addition, pembrolizumab improved event-free survival (EFS) (HR 0.63; 95% CI 0.43–0.93). Grade 3/4 toxicities also occurred more frequently with pembrolizumab (78% vs. 73%). Recently, an updated version of this study was presented at the Annual Meeting of the European Society of Medical Oncology [82]. The authors confirmed improved EFS (84.5% vs. 76.8%) and reported a strong trend towards longer OS (HR 0.72; 95% CI 0.51–1.02).

The NeoTRIPaPDL1 Michelangelo randomized trial evaluated neoadjuvant nab-paclitaxel treatment with or without atezolizumab in triple-negative, early high-risk breast cancer and locally advanced breast cancer and failed to demonstrate a significant increase in pCR with atezolizumab [83]. Recently, however, results were presented on the efficacy and safety of atezolizumab compared with a placebo in combination with nab-paclitaxel followed by doxorubicin plus cyclophosphamide as a neoadjuvant treatment of early TNBC [84]. In total, 333 patients with early-stage TNBC were enrolled in the double-blind, randomized phase III IMpassion031 trial. Atezolizumab increased pCR from 41% to 58%. In the PD-L1-positive population, pCR was increased from 49% to 69%. Treatment-related serious adverse events occurred in 23% and 16% of cases, respectively. The authors concluded that neoadjuvant treatment with atezolizumab in combination with nab-paclitaxel and anthracycline-based chemotherapy improves pCR in early-stage TNBC patients and has an acceptable safety profile. The results of the phase III trials in early-stage TNBC are summarized in Table 4:

Indeed, the vast majority of randomized trials using ICPi in early or advanced TNBC showed significant benefits over standard therapy alone. When combined with an acceptable safety profile, immune checkpoint inhibitors are a promising new therapeutic option in TNBC. Recently, the Society for Immunotherapy of Cancer (SITC) published a clinical practice guideline on immunotherapy for breast cancer [85]. Recommendations in this clinical practice guideline include diagnostic testing, treatment planning, immune-related adverse events, and patient quality of life considerations to provide guidance to the oncology community treating breast cancer patients with immunotherapies.

## 5. Predictive Markers for Immune Checkpoint Inhibitors

Currently, the only established predictive biomarker for response to ICPi in advanced TNBC is PD-L1 status. Recent analyses have shown a potential role of TMB in response to durvalumab in early TNBC [77]. In a recently published comprehensive genomic analysis of 3831 consecutive breast cancer samples, potential biomarkers (e.g., TMB, microsatellite instability [MSI], BRCA mutations) were assessed to guide the use of ICPIs in these patients [86]. Interferon-γ (IFN-γ) plays a crucial role in the regulation of anti-tumor immunity [87]. Upon ligand binding, IFN-y receptor 1 and 2 (IFNγR1 and IFNγR2) oligomerize and transphosphorylate, activating Janus-activated kinase (JAK) 1 and 2. Thereby, IFNγR1 is phosphorylated, creating a docking site for the signal transducer and activator of transcription (STAT) 1. Interferon-γ (IFN-γ) signaling signatures are associated with clinical response to treatment with ICPi [88]. Similarly, JAK/STAT pathways predict response to ICPi therapy [89]. In addition, cancer stem cells are a potential biomarker to predict the effectiviness of ICPis [90]. However, for all of these potential biomarkers, prospective randomized trials are needed to assess the predictive value in response to immune checkpoint inhibitors.

## 6. Adverse Events with Immune Checkpoint Inhibitors

Adverse effects of ICPis are mainly explained by their mode of action. ICPis block so-called immune checkpoints, which act as “brakes” for triggered immune reactions. When this “brake” is blocked by antibodies, such as ICPis, an unrestrained immune response can occur, which can also attack the body’s own tissues through autoimmune phenomena. This is associated with a spectrum of side effects related to the mechanism of action. The side effects can affect multiple organs of the body and most commonly occur in the skin, gastrointestinal tract, lungs, thyroid, adrenal, pituitary, kidney, nervous system, musculoskeletal system, eyes, or cardiovascular system. In addition to organ-specific side effects, infusion-related reactions such as fever, chills, shortness of breath, and sudden redness of the face, neck, or chest may occur [91,92,93] (Table 5). During treatment, it is important to note that these immune-mediated side effects may occur at widely varying intervals and sometimes even after cessation of therapy with ICPi.

### Adverse Event Management

Early diagnosis and therapy can reduce the severity and duration of immune-mediated adverse events. Proper management of these adverse events is, therefore, crucial. Depending on the severity of the side effects, different therapeutic measures are recommended. In case of marked worsening of symptoms, therapy with corticosteroids or even discontinuation of treatment with ICPi is required. (Table 6) [91].

## 7. Vaccination

The idea of using vaccines to boost the immune system against breast cancer has a long history [94]. Especially in cold tumors, there are few or no tumor-infiltrating lymphocytes. Possible reasons for this could be a lack of tumor antigens, a defect in antigen presentation, a lack of T-cell activation, and a deficit in homing to the tumor bed [95]. Vaccines may help to overcome this problem by improving the presentation of tumor-associated antigens, leading to a better immune response. Indeed, enhancing antigen presentation through vaccination is an obvious way to elicit a protective immune response against breast cancer. However, vaccination against solid tumors, such as breast cancer, has shown limited efficacy. In most cases, known antigens, such as HER2, have been used, but these vaccination strategies have shown only modest efficacy in breast cancer patients (Table 7) [96,97,98,99].

However, a fundamental drawback of this approach is that immune responses against known self-antigens, such as HER2, are usually weak because T lymphocytes, which have a high affinity for these self-antigens, are subject to central tolerance. With the help of high-throughput mutation analysis techniques, such as next generation sequencing (NGS), single non-synonymous somatic mutations (the so-called mutanome) are increasingly coming into focus [100]. The resulting neoantigens are ideal for individual vaccination. Using complex computational prediction algorithms, the neoantigens with the highest expected immunogenicity are selected from the mutanome of a tumor. The mRNA of these neoantigens is then used as a vaccine [101]. The mRNAs are administered intravenously as a nano-particulate lipoplex formulation and are selectively delivered to splenic antigen-presenting cells. The encoded antigens are translated into proteins that are rapidly processed and presented as peptides on the surface of APCs, which, in turn, leads to the induction of antigen-specific T-cell responses [102].

While the focus of anti-tumor immunity research has long been on MHC I and CD8 T cells, it has been shown in mouse models that the majority of immunogenic mutations are presented via MHC II and recognized by CD4 T cells [103]. Meanwhile, the clinical efficacy of individual RNA vaccination against the individual mutanoma of a tumor in patients with advanced malignant melanome has been described [104].

Based on these encouraging results, the Mutanome Engineered RNA Immuno-Therapy (MERIT) project, a collaborative effort of partners from academia and industry, funded by the European Union’s Seventh Framework Programme (FP7), aims to clinically and industrially validate a pioneering mRNA-based immunotherapy concept that targets individually expressed tumor antigens and tumor-specific mutations in patients with early TNBC. [102]. Before treatment, every patient’s tumor will be profiled to select suitable shared tumor antigens (MERIT WAREHOUSE) and identify individual mutations (MERIT MUTANOME). Ideally, this approach will lead to a paradigm shift from stratified therapy targeting single common biomarkers, such as HER2, to fully individualized treatment targeting patient-specific mutations. As an integral part of the MERIT project, we have initiated a phase I study in early TNBC after completion of standard (neo)adjuvant chemotherapy [105]. TNBC-MERIT is a phase I study (NCT02316457) evaluating the feasibility, safety, and immunogenicity of a lipoplex-formulated intravenous mRNA vaccine encoding different tumor antigens in TNBC patients after surgery and (neo-)adjuvant chemotherapy. Patients in two arms of this study received a personalized set of pre-formulated, non-mutated, shared tumor-associated antigens (MERIT WAREHOUSE) with or without universal T helper epitopes. In a third arm, patients were inoculated with IVAC_M_uID, an on-demand individualized neoantigen-specific immunotherapy (iNeST) encoding neoepitopes derived from up to 20 cancer mutations identified by NGS (MERIT MUTANOME). The objective of this study was to demonstrate the feasibility, safety, and biological efficacy of a llipoplex-formulated intavenous mRNA vaccine encoding different tumor antigens. Patients received eight intravenous vaccinations with either a personalized mRNA vaccine based on the antigen expression profile of each tumor (MERIT WAREHOUSE) or an individualized mRNA vaccine against up to 20 neoepitopes identified by NGS (MERIT MUTANOME). Recently, at the Annual Meeting of the European Society of Medical Oncology, we reported preliminary immune response data in IVAC_M_uID-vaccinated patients analyzed by interferon-γ enzyme-linked-immuno-spot (IFNγ-ELISpot), T-cell receptor (TCR) profiling, and single-cell TCR sequencing [106]. Immunogenicity data were obtained from all 14 patients treated with IVAC_M_uID. All patients studied had vaccine-induced CD4+ and/or CD8+ T-cell responses against 1 to 10 of the vaccine neoepitopes detected by IFNγ-ELISpot ex vivo or after in vitro stimulation. A substantial number of T-cell responses against individual neoepitopes were induced de novo, of a high magnitude, and durable. One of the index patients characterized in more detail had CD4+ and/or CD8+ T-cell responses against 10 of 20 vaccine neoepitopes. The highly poly-epitopic TCR-clonotype diversified CD8+ T-cell response comprised, in aggregate, about 30% of total peripheral CD8+ T cells and was sustained at high levels for more than 600 days after the last vaccination. This suggests that the individualized neoantigen-specific vaccine is highly efficient in inducing strong polyepitopic T-cell responses in patients with TNBC in the post-(neo)adjuvant phase. As an important effector cytokine for cancer immunity, IFNγ also has prognostic and predictive significance in basal-like or triple-negative breast cancer, arguing for a protective effect of IFNγ-mediated immune responses by vaccination. [107,108].

With this vaccination strategy, T-cell responses against tumor-specific neoantigens can be induced. Such vaccines may lead to an increase in the immunogenicity of tumors that lack spontaneous immunogenicity, which should make these tumors more responsive to treatment with ICPis. Therefore, a combination of RNA vaccination and ICPis may be useful to stimulate the endogenous immune system against tumor cells, including in patients with prior ICPi experience [109]. Sahin and coworkers reported in this exploratory interim analysis of a phase I study that RNA vaccination is an effective immunotherapy in patients with ICPi-experienced melanoma, resulting in durable objective responses accompanied by the induction of strong CD4+ and CD8+ T-cell immunity to the vaccine antigens.

## 8. Conclusions

The immune system plays an important role in breast cancer. High expression of tumor-infiltrating lymphocytes or immune transcripts is associated with improved prognosis, as well as enhanced response to chemotherapy, especially in TNBC. Novel therapies, such as immune checkpoint inhibitors, have improved survival in triple-negative breast cancer. In addition, individualized vaccination strategies using mRNA vaccines against mutant tumor antigens are promising. Thus, a potentially rewarding future direction in the treatment of triple-negative breast cancer may be the combination of personalized vaccination and immune checkpoint inhibitors to fully harness the power of anti-tumor immunity.

## Figures and Tables

**Table 1:** Tumor-infiltrating lymphocytes in early triple-negative breast cancer.

Author	*n*	pCR	DDFS	DFS	OS
Denkert et al., 2015 [30]	314	33.8% vs. 59.9% *p* = 0.004	-	-	-
Denkert et al., 2018 [31]	906	11% vs. 50% *p* < 0.0001	-	HR 0.93 (0.87–0.98)	HR 0.92 (0.86–0.99)
Loi et al., 2014 [32]	134	-	HR 0.77 (0.61–0.98)		-
Adams et al., 2014 [34]	506	-	-	HR 0.86 (0.76–0.98)	HR 0.82 (0.68–0.99)
Hida et al., 2019 [37]	234	21% vs. 46% *p* = 0.032	-	HR 3.71 (1.60–8.57)	HR 3.87 (1.46–10.27)
Ibrahim et al., 2014 [36]	2987	-	HR 0.78 (0.68–0.90)	HR 0.70 (0.56–0.87)	HR 0.66 (0.53–0.83)
Loi et al., 2013 [35]	512	-	.	HR 0.31 (0.11–0.84)	HR 0.30 (0.094–0.95)

Abbreviations: DDFS, distant disease-free survival; DFS, disease-free survival; HR, hazard ratio; OS, overall survival; pCR, pathologic complete response (low vs. high tumor-infiltating lymphocytes); TNBC, triple-negative breast cancer; vs., versus.

**Table 3:** Randomized evidence for ICPi in advanced breast cancer.

Author	*n*	Therapy	PFS in ITT	OS in ITT
Schmid et al., 2018 [70]	902	Nab-paclitaxel +/− atezolizumab	7.2 vs. 5.5 m HR 0.80 (0.69–0.92)	21.3 vs. 17.6 m HR 0.84 (0.69–1.02)
Miles et al., 2021 [72]	651	Paclitaxel +/− atezolizumab	6.0 vs. 5.7 m HR 0.82 (0.60–1.12)	22.1 vs. 28.3 m HR 1.11 (0.76–1.64)
Cortes et al., 2020 [74]	847	Chemotherapy +/− pembrolizumab	7.5 vs. 5.6 m HR 0.82 (0.69–0.97)	
Winer et al., 2021 [58]	622	Pembrolizumab vs. chemotherapy		10.7 vs.10.2 m HR 0.86 (0.69–1.06)
Tolaney et al., 2020 [75]	88	Eribulin +/− pembrolizumab	4.1 vs. 4.2 m HR 0.80 (0.50–1.26)	13.4 vs. 12.5 m HR 0.87 (0.48–1.59)

Abbreviations: HR, hazard ratio; ICPis, immune checkpoint inhibitors; ITT, intention to treat; m, months; OS, overall survival; PFS, progression-free survival; vs., versus.

**Table 4:** Randomized evidence for ICPis in early triple-negative breast cancer.

Author	*n*	Therapy	pCR in ITT	EFS in ITT	OS in ITT
Schmid et al., 2020 [81]; ESMO 2021 [82]	1174	Chemotherapy +/− pembrolizumab	64.8% vs. 51.2%	HR 0.63 0.43–0.93)	HR 0.72 (0.51–1.02)
Mittendorf et al., 2020 [84]	333	Chemotherapy +/− atezolizumab	58% vs. 41	HR 0.76 (0.40–1.44)	
Gianni et al., SABCS 2019 [83]	280	Chemotherapy +/− atezolizumab	43.5% vs. 40.8%		
Loibl et al., 2019 [76]; ASCO 2021 [79]	174	Chemotherapy +/− durvalumab	53.4 vs. 44.2		HR 0.26 (0.09–0.79)

Abbreviations: EFS, event-free survival; ESMO, European Society of Medical Oncology; HR, hazard ratio; ICPis, immune checkpoint inhibitors; ITT, intention to treat; m, months; OS, overall survival; pCR, pathologic complete response; SABCS, San Antonio Breast Cancer Symposium; vs., versus.

**Table 5:** Immune-mediated adverse effects of immune checkpoint inhibitors.

Organ	Occurrence with PD-(L)1 Inhibitors	Symptoms
Brain	Encephalitis < 1% Aseptic meningitis < 0.5%	Headache, changes in mental status, confusion, depressed mood, sensitivity to light, seizures, motor or sensory disturbances, meningitis, neck stiffness.
Nerves	Guillain- Barré Syndrome < 0.5% Peripheral neuropathy 16%	Muscle weakness (including eye muscles), fatigue, difficulty swallowing, paresthesia or altered sensory perception, ascending or progressive paralysis, weakness of respiratory muscles.
Skin	Rash 10% Pruritus 15% [93]	Persistent and/or severe skin rash or itching
Thyroid gland, adrenal glands, pituitary gland, islets of Langerhans of the pancreas	Hypothyroidism 8% Hyperthroidism 5% Adrenal insufficiency 1% Hypophysitis 1% Diabetes mellitus 1%	Fatigue, headache, changes in mental status, intolerance to heat or cold, tachycardia or bradycardia, irregularity in bowel movements, weight change, polyuria or polydipsia, blurred vision.
Lung	Pneumonitis 4%	Difficulty breathing or coughing, radiographic changes (e.g., focal morning opacity, patchy, patchy infiltrates), dyspnea, hypoxia
Liver	Hepatitis 5%	Increase in transaminases, increase in total bilirubin, jaundice, right-sided abdominal pain, fatigue.
Pancreas	Pancreatitis 3%	Abdominal pain, nausea, vomiting, and fever
Intestine	Diarrhea/colitis 11%	Watery, soft, or liquid stools; diarrhea; abdominal pain; mucus or blood in the stool.

**Table 6:** Treatment of adverse effects of immune checkpoint inhibitors.

CTC	Actions
I	- Continue with close monitoring
II	- Discontinue therapy until improvement to grade 1, consider corticosteroids if necessary
III	- Administration of corticosteroids (prednisone 1 to 2 mg/kg/d or methylprednisolone 1 to 2 mg/kg/d) - Taper off 4–6 weeks - Infliximab, if no improvement under corticosteroids within 48–72 h
IV	- Termination of therapy (exception: endocrinopathies with improvement through hormone substitution)

CTC: common criteria of toxicity.

**Table 7:** Vaccination trials in breast cancer.

Author	*n*	Setting	Vaccine	PFS	DFS
Peoples et al., 2005 [96]	53	Adjuvant	E75 + GM-CSF	-	85.7% vs. 59.8%
Disis et al., 2009 [99]	22	Advanced HER2+	HER2 Th peptide + trastuzumab	17.7 months	-
Mittendorf et al., 2014 [98]	187	Adjuvant	E75 + GM-CSF	-	89.7% vs. 80.2% *p* = 0.08
Mittendorf et al., 2016 [97]	298	Adjuvant	AE37 + GM-CSF	-	80.8% vs. 79.5% *p* = 0.70

Abbreviations: DFS, disease-free survival; GM-CSF, granulocyte macrophage colony-stimulating factor; PFS, progression-free survival; Th, T helper; vs., versus.
